# Harnessing machine learning and AI-driven analytics to identify novel drug targets and predict chemotherapy efficacy in NSCLC

**DOI:** 10.3389/fphar.2025.1555040

**Published:** 2025-02-19

**Authors:** Shaojia Qin, Biyu Deng, Dan Mo, Zhengyou Zhang, Xuan Wei, Zhougui Ling

**Affiliations:** ^1^ Department of Pulmonary and Critical Care Medicine, Laibin People’s Hospital, Laibin, China; ^2^ Department of Pulmonary and Critical Care Medicine, The Fourth Affiliated Hospital of Guangxi Medical University, Liuzhou, China

**Keywords:** non-small cell lung cancer (NSCLC), chemotherapy response, mitochondria-derived RNAs (mtRNAs), BiomedGPT, machine learning, artificial intelligence, biomarker discovery

## Abstract

**Introduction:**

Non-small cell lung cancer (NSCLC) constitutes the majority of lung cancer cases and exhibits marked heterogeneity in both clinical presentation and molecular profiles, leading to variable responses to chemotherapy. Emerging evidence suggests that mitochondria-derived RNAs (mtRNAs) may serve as novel biomarkers, although their role in predicting chemotherapy outcomes remains to be fully explored.

**Methods:**

In this study, peripheral blood mononuclear cells were obtained from NSCLC patients for analysis of the mtRNA ratio (mt_tRNA-Tyr-GTA_5_end to mt_tRNA-Phe-GAA), while thoracic CT images were processed to derive an AI-driven BiomedGPT variable. Although individual clinical factors (Sex, Age, History_of_smoking, Pathological_type, Stage) offered limited predictive power when used in isolation, their integration into a random forest model improved sensitivity in the training set, albeit with reduced generalizability in the validation cohort. The subsequent integration of the BiomedGPT score and mtRNA ratio significantly enhanced predictive performance across both training and validation datasets.

**Results:**

An all-inclusive model combining clinical data, AI-derived variables, and mtRNA biomarkers produced a risk score capable of discriminating patients into high- and low-risk groups for progression-free survival and overall survival, with statistically significant differences observed between these groups.

**Discussion:**

These findings highlight the potential of integrating mtRNA biomarkers with advanced AI methods to refine therapeutic decision-making in NSCLC, underscoring the importance of combining diverse data sources in precision oncology.

## Introduction

Non-small cell lung cancer (NSCLC) remains a global health challenge, constituting approximately 85% of all lung cancer cases and exhibiting extensive heterogeneity both clinically and molecularly (Pathak et al., 2004; Chen et al., 2024). Although targeted therapies, immunotherapeutic agents, and improved surgical techniques have enhanced patient outcomes, systemic chemotherapy remains a key component for many individuals with advanced disease (Tan et al., 2016). Nonetheless, responses to chemotherapeutic regimens vary widely, reflecting the need to identify biomarkers capable of predicting patient-specific efficacy and to discover novel drug targets that could refine treatment strategies (Stewart et al., 2007). Traditional molecular biology approaches—employing high-throughput omics (e.g., transcriptomics, proteomics) and pathway-focused interrogation—have robustly identified important oncogenic drivers and prognostic indicators in NSCLC. However, these methods alone often struggle to capture the intricate interplay among diverse molecular and clinical variables, especially as the volume and complexity of biological data continue to escalate.

In parallel, mitochondria-derived RNAs (mtRNAs) have garnered increasing attention as potential biomarkers and therapeutic leads in cancer (Shafiei Asheghabadi et al., 2024). Mitochondria are central to cellular metabolism, apoptosis, and oxidative stress, rendering their genetic and transcriptomic signatures highly relevant to tumor initiation and progression. Mitochondria-derived RNAs (mtRNAs) have shown promise in oncology, with recent studies highlighting their diagnostic value and potential role in predicting treatment outcomes. Peripheral blood mononuclear cells (PBMCs), serve as a minimally invasive source for capturing systemic biomarkers associated with immune and metabolic modulations influenced by the tumor microenvironment. This allows for dynamic longitudinal monitoring, enabling the exploration of mtRNA ratios as predictors of chemotherapy response. Our previous study highlighted the diagnostic potential of mitochondria-derived RNAs (mtRNAs) in lung cancer by employing a ratio-based expression framework, specifically focusing on mt-tRNA-Tyr-GTA-5 and mt-tRNA-Phe-GAA, to enhance detection accuracy (Yu et al., 2023). These mtRNAs have demonstrated significant promise as diagnostic markers and potential therapeutic targets due to their involvement in mitochondrial function and cancer progression. However, despite their established roles, their ability to predict chemotherapy resistance and clinical outcomes has not been explored. Understanding the prognostic value of these mtRNA ratios could provide critical insights into mechanisms of chemotherapy resistance, offering new avenues for precision medicine in non-small cell lung cancer (NSCLC).

Given the multifaceted nature of lung tumors, artificial intelligence (AI) and machine learning (ML) techniques offer powerful tools for data integration and hypothesis generation (Ahmed et al., 2020). By rapidly processing and extracting insights from large-scale multi-omic datasets, AI-driven models expedite the discovery of new drug targets and facilitate refined prognostic modeling in oncology (Biswas and Chakrabarti, 2020). Yet such methodologies are not without limitations, including concerns about reproducibility, model interpretability, and clinical validation. To address these challenges, hybrid approaches that combine the mechanistic rigor of conventional experimental methods with the pattern-recognition capabilities of AI can capitalize on the strengths of each paradigm (Țîrcovnicu and Hațegan, 2023). Classical machine learning algorithms, such as logistic regression or random forest, deliver interpretable outputs and straightforward implementations, whereas deep learning architectures enable sophisticated mapping of high-dimensional, heterogeneous data (Pedro, 2023; Onoja, 2023).

Within the realm of deep learning, transformer-based architectures—notably those employing attention mechanisms—have demonstrated remarkable success in language modeling, natural language processing, and complex sequence analysis (Singh and Mahmood, 2021). Leveraging self-attention, these models excel at identifying long-range dependencies within the data, making them ideal for biomedical tasks involving large transcriptomic or proteomic datasets. BiomedGPT, a domain-tailored evolution of the GPT (Generative Pretrained Transformer) family, builds on these breakthroughs by integrating an expansive corpus of clinical, molecular, and textual data (Zhang et al., 2023). Through transfer learning and specialized fine-tuning, BiomedGPT is particularly adept at analyzing multi-modal inputs to uncover subtle gene expression patterns that may correspond to chemotherapeutic response, thereby augmenting the accuracy and speed of biomarker identification relative to classic pipelines.

In this study, we harness both machine learning and AI-driven analytics—exemplified by BiomedGPT—to explore the role of mtRNAs, initially shown to hold diagnostic value in lung cancer, for their previously untested ability to predict chemotherapy outcomes in NSCLC and identify additional novel drug targets associated with patient response. By comparing the performance of transformer-based analytics against conventional ML models, we aim to elucidate any reproducibility issues, address interpretability constraints, and validate predictions using independent cohorts. Ultimately, we seek to demonstrate how the analysis of mtRNA expression signatures, integrated into a broader AI pipeline, enhances our capacity to predict chemotherapy efficacy in NSCLC, facilitating precision oncology approaches that may improve patient stratification and outcomes.

## Methods

### Patient enrollment and sample collection

Patients with advanced non-small cell lung cancer (NSCLC) scheduled to receive standard chemotherapy were prospectively recruited between January 2020 and December 2022 from the Department of Oncology at the Fourth Affiliated Hospital of Guangxi Medical University and Laibin People’s Hospital. Chemotherapy regimens followed institutional protocols and adhered to standard guidelines for NSCLC. Patients received cisplatin or carboplatin, combined with either paclitaxel, gemcitabine, or pemetrexed as the second agent. These regimens were administered every 21 days for up to four to six cycles, consistent with established clinical practice and evidence-based guidelines (Scagliotti et al., 2008; Schiller et al., 2002).Inclusion criteria comprised: age ≥18 years, histologically confirmed NSCLC (stage IIIB, IIIC, or IV), measurable lesions on CT imaging, and an Eastern Cooperative Oncology Group (ECOG) performance status of 0–2. Exclusion criteria included prior systemic therapy within 3 months, concomitant malignancies, significant cardiopulmonary comorbidities, or refusal to provide written informed consent. All patients provided peripheral blood samples prior to their first chemotherapy cycle; peripheral blood mononuclear cells (PBMCs) were isolated by density-gradient centrifugation using Ficoll-Paque PLUS (Cytiva) and stored in RNAlater Stabilization Solution (Invitrogen) at −80°C until analysis. Chemotherapy regimens were administered per institutional protocols (commonly platinum-doublet therapies), and response was determined after two treatment cycles via Response Evaluation Criteria in Solid Tumors (RECIST) version 1.1. Patients classified as having complete or partial responses were assigned to the responder group, while stable or progressive disease indicated non-response. Ethical approval for this study was granted by the Institutional Review Board of the Fourth Affiliated Hospital of Guangxi Medical University (KY2023329), and all participants provided written informed consent according to the Declaration of Helsinki.

### BiomedGPT fine-tuning and CT image analysis

Thoracic CT scans were obtained from all enrolled subjects at baseline for diagnostic assessment. Imaging protocols varied slightly but generally included spiral CT with slice thickness of ≤5 mm. DICOM data were anonymized and processed using Python 3.9 and OpenCV (version 4.5) to ensure consistent resolution and contrast normalization.

The full CT scans were systematically divided into smaller non-overlapping tiles of 224 × 224 pixels to match the input dimensions required by BiomedGPT, facilitating uniform data processing. This tiling process allowed the model to analyze localized radiomic features across the entire lung field, capturing variations in tumor size, shape, texture, and spatial heterogeneity. These features are critical for predicting treatment responses and progression-free survival (PFS), as they reflect underlying tumor biology and phenotypic diversity. BiomedGPT—a domain-specific Generative Pretrained Transformer—was initially pretrained on large-scale biomedical text corpora and further fine-tuned on a dedicated archive of lung cancer CT scans curated. The fine-tuning phase involved 86^−ΔΔCT^ images from archival datasets, each labeled with a corresponding clinical outcome (response vs. non-response). During this step, the final layer of BiomedGPT was replaced with a custom classification head, and the network’s learning rate was set to 1e-5, with batch size 8. Training continued for 20 epochs or until validation loss plateaued. Model outputs were then distilled into a single numeric probability indicating the likelihood of chemotherapy response. These response probabilities were used as the “AI” variable for subsequent machine learning integration.

### RNA isolation and quantitative PCR

Total RNA was extracted from PBMC samples using TRIzol reagent (Thermo Fisher Scientific) following the manufacturer’s instructions. RNA concentration and purity were assessed by NanoDrop 2000 (Thermo Fisher Scientific), ensuring an A260/A280 ratio between 1.8 and 2.0. For reverse transcription, 1 μg of total RNA was used with the High-Capacity cDNA Reverse Transcription Kit (Applied Biosystems). The mtRNA ratio of interest—mt_tRNA-Tyr-GTA_5_end to mt_tRNA-Phe-GAA—was quantified by quantitative PCR on a QuantStudio 3 Real-Time PCR System (Applied Biosystems). Primers were designed using Primer-BLAST (NCBI) and synthesized by Integrated DNA Technologies (IDT). Each reaction (10 μL total volume) contained 5 μL of PowerUp SYBR Green Master Mix (Applied Biosystems), 0.4 μM of forward and reverse primers, and 1 μL of cDNA template. PCR cycling parameters were 95°C for 2 min, followed by 40 cycles of 95°C for 15 s and 60°C for 1 min. Relative expression levels were computed by the 2^(-ΔCt) method, using β-actin as the endogenous control. For ratio calculation, mt_tRNA-Tyr-GTA_5_end expression was normalized to that of mt_tRNA-Phe-GAA.

### Machine learning model development

Clinical variables (Sex, Age, History_of_smoking, Pathological_type, and Stage) were first evaluated individually to predict chemotherapy response using logistic regression implemented in Python’s scikit-learn (version 1.0). A random forest classifier (1000 decision trees, Gini impurity as splitting criterion) was then constructed to integrate these five features into a combined clinical model. Separately, two single-factor models were built for the AI output (BiomedGPT response probability) and for the mtRNA ratio (mt_tRNA-Tyr-GTA_5_end/mt_tRNA-Phe-GAA). Next, these two features (AI and mtRNA) were merged into a random forest (Model A). An expanded model (Model B) included the same two variables plus the five clinical factors. Hyperparameters (e.g., tree depth, minimum samples split) were tuned via 5-fold cross-validation on the training set. Probabilistic outputs (predict_proba) of each model were used to compute the area under the ROC curve (AUC). Finally, the best-performing integrated model’s output was converted into a continuous risk score for downstream survival analysis.

### Statistical analyses

All data processing and statistical evaluations were carried out using R (version 4.1.2) and Python (version 3.9) (Guijarro, 2023). Continuous variables were summarized as medians and interquartile ranges; categorical data were expressed as frequencies or percentages. Differences in predictive performance were assessed by AUC comparisons via the DeLong test, considering p < 0.05 as statistically significant. Kaplan-Meier survival analyses were conducted with the survival package (R), dividing subjects into high- or low-risk groups based on the integrated model’s median risk score (Therneau and Lumley, 2013). Log-rank tests determined the significance of survival differences between groups; p-values below 0.05 were interpreted as significant. Figures were generated in matplotlib (Python). Where relevant, p-values were adjusted for multiple comparisons using the Benjamini–Hochberg procedure.

## Results

### Clinical factor analysis

**FIGURE 1:**
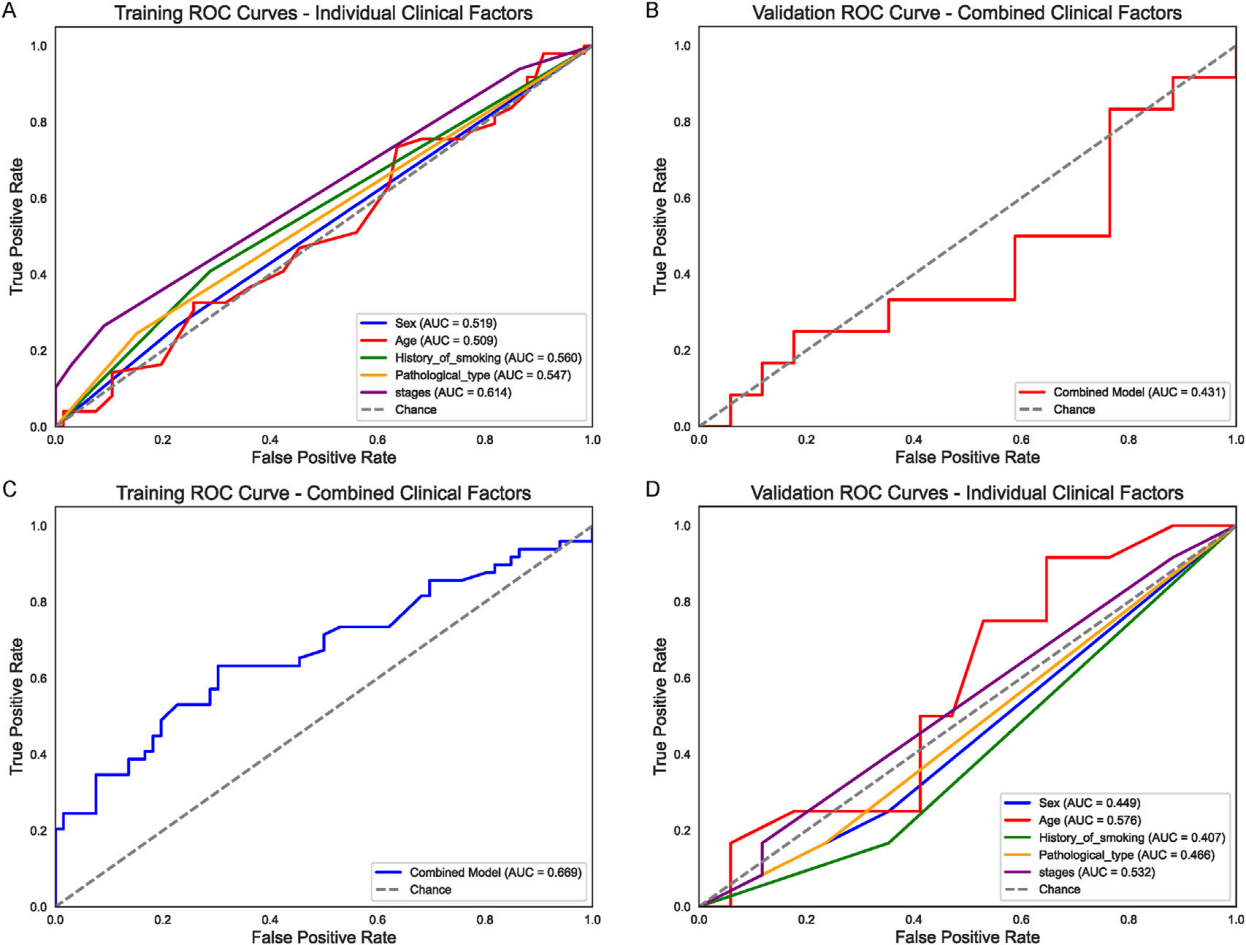
Clinical Factor Analysis for Chemotherapy Response **(A)** Receiver operating characteristic (ROC) curves for individual clinical factors (Sex, Age, History_of_smoking, Pathological_type, and Stage) in the training set. Stage shows the highest area under the curve (AUC = 0.614), followed by History_of_smoking (AUC = 0.560). **(B)** Corresponding ROC curves in the validation set, where none of the single-factor models exceed an AUC of 0.576. **(C)** ROC curve of the random forest “Combined Clinical Model” in the training cohort (AUC = 0.669). **(D)** Validation set performance of the combined clinical model (AUC = 0.431), indicating limited generalizability of clinical-only features.

A total of 86 patients (19 female, 67 male) were assigned to the training set, and 58 patients (18 female, 40 male) to the validation set. All individuals underwent chemotherapy, with median ages of 63 and 64 years, respectively (training range 37–82; validation range 49–87). Baseline clinical characteristics, including smoking history, tumor histology, and disease stage, are summarized in the Table. We first assessed the predictive value of individual clinical factors—Sex, Age, History of smoking, Pathological type, and Stage for chemotherapy response in non-small cell lung cancer (NSCLC). In the training set (Figure 1A), Stage showed the highest area under the curve (AUC = 0.614), followed by History_of_smoking (AUC = 0.560), while the remaining factors exhibited relatively modest performance (AUC range 0.509–0.547). When applied to the validation cohort (Figure 1D), none of these single-factor models exceeded an AUC of 0.576. Subsequently, a random forest classifier combining all five clinical variables (“Combined Model”) achieved an AUC of 0.669 in the training cohort (Figure 1C), but only 0.431 in the validation set (Figure 1B), indicating limited generalizability using clinical features alone.

### AI and mtRNA ratio as independent predictors

**FIGURE 2:**
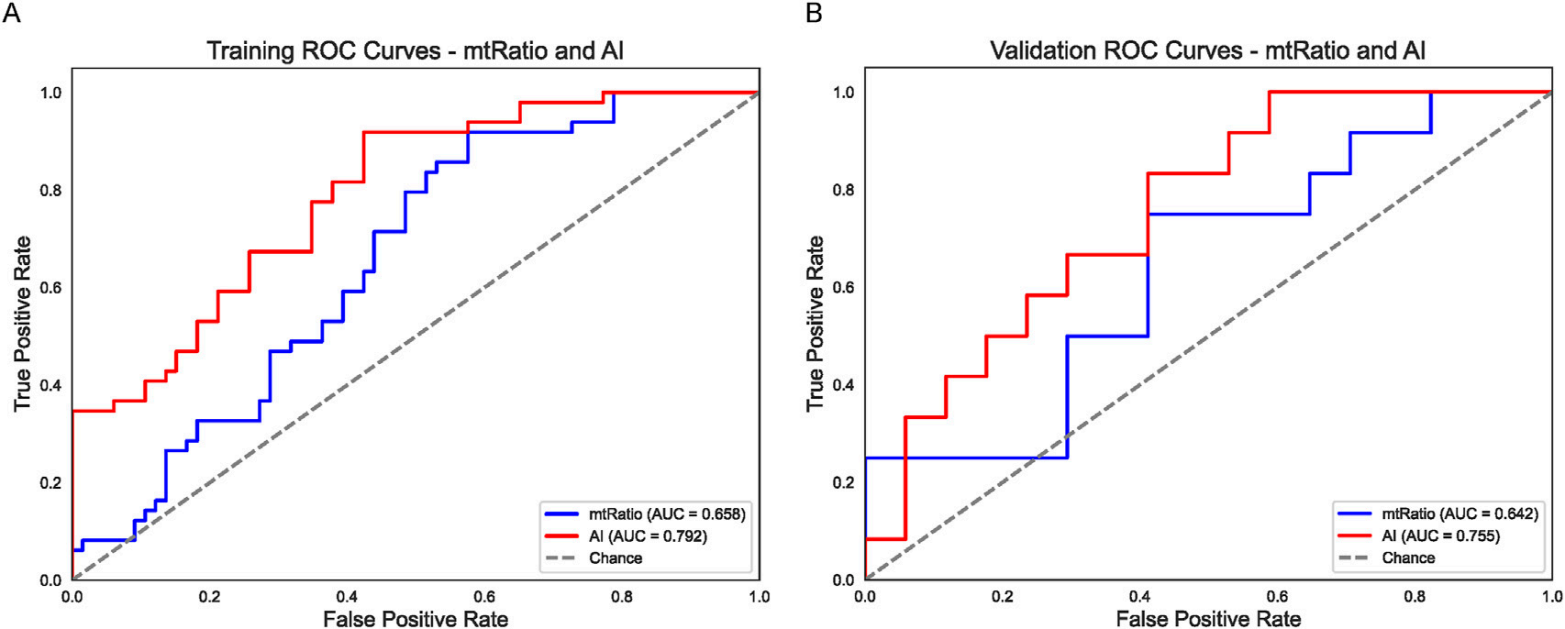
AI (BiomedGPT) and mtRNA Ratio as Independent Predictors **(A)** ROC curves in the training set for the BiomedGPT-based AI variable (red line, AUC = 0.792) and the mtRNA ratio (blue line, AUC = 0.658). **(B)** Validation set ROC curves illustrate a similar trend, with the AI predictor (red line, AUC = 0.755) outperforming the mtRNA ratio (blue line, AUC = 0.642). Both strategies exceed the accuracy of most single-factor clinical models shown in Figure 1.

Next, we evaluated whether an AI-based metric derived from BiomedGPT’s analysis of CT images (“AI”) and a novel mtRNA ratio (mt_tRNA-Tyr-GTA_5_end/mt_tRNA-Phe-GAA) could improve prediction of chemotherapy outcomes. As shown in Figure 2A, the mtRNA ratio alone (blue line) achieved an AUC of 0.658 in the training set, whereas the AI predictor (red line) reached 0.792. Similar findings emerged in the validation cohort (Figure 2B), with AUCs of 0.642 (mtRNA ratio) and 0.755 (AI). Both metrics outperformed most individual clinical factors, suggesting that AI-derived features and mtRNA-based biomarkers capture additional biologically relevant signals not accounted for by standard clinical data.

## Integrated models combining AI, mtRNA ratio, and clinical information

**FIGURE 3:**
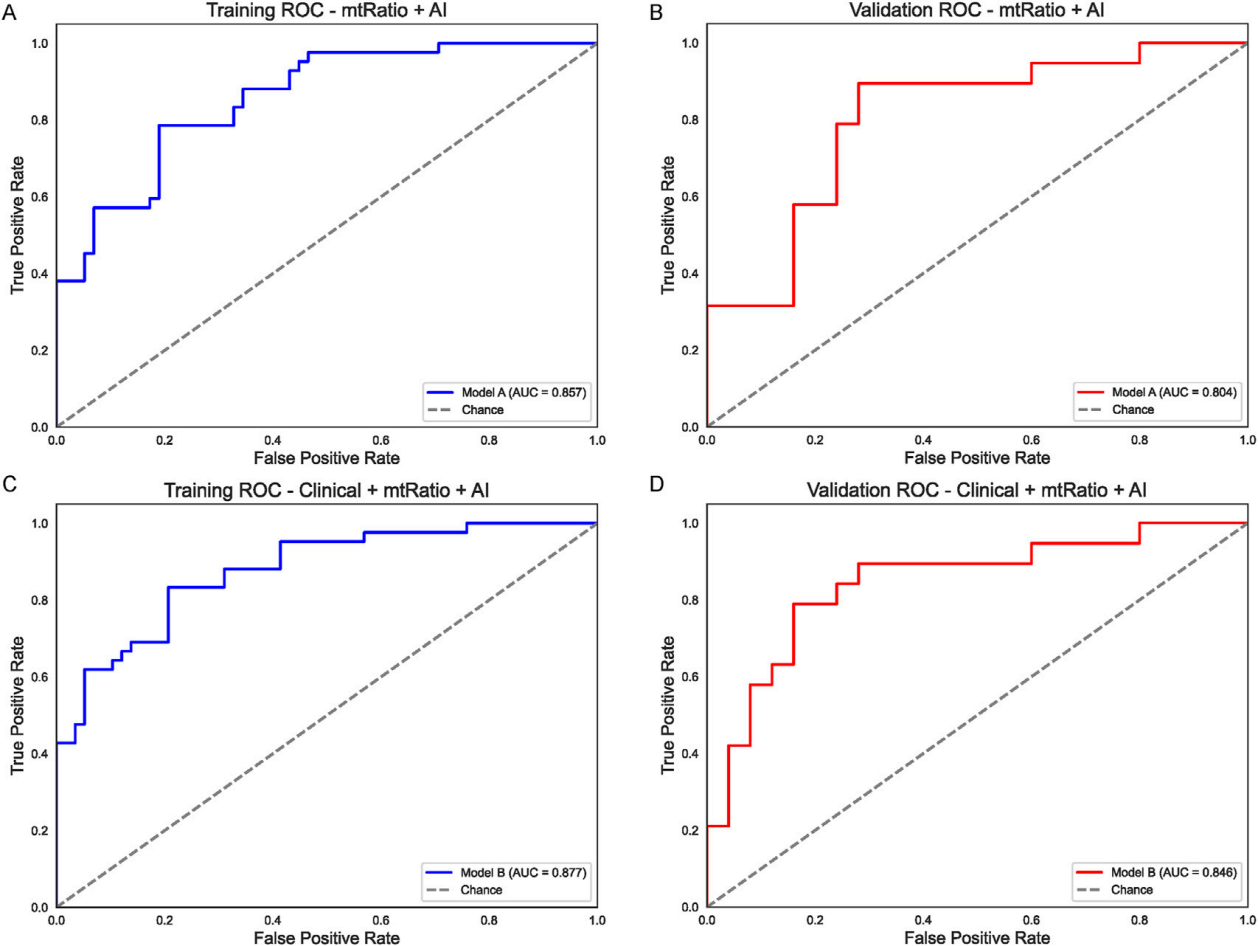
Integrated Models Combining AI, mtRNA Ratio, and Clinical Factors **(A)** Performance of Model A (AI + mtRNA) in the training set (AUC = 0.857). **(B)** Model A retains strong predictive power in the validation set (AUC = 0.804). **(C)** Model B (AI + mtRNA +5 clinical variables) achieves a higher AUC of 0.877 in the training cohort, indicating synergistic effects of integrating multiple predictors. **(D)** Validation set performance of Model B (AUC = 0.846) confirms improved generalization compared to clinical-only models.

To capitalize on these complementary predictors, a random forest approach was employed to merge the AI output (considered as a continuous numeric variable) and the mtRNA ratio into a single model (Model A). In the training group (Figure 3A), Model A yielded an AUC of 0.857, and its validation performance remained strong at 0.804 (Figure 3B). Building on this, a more comprehensive model (Model B) included the same two factors plus the five clinical variables. As shown in Figure 3C, the resulting training-set AUC for Model B rose to 0.877, while the validation-set AUC reached 0.846 (Figure 3D). These integrated strategies substantially outperformed the clinical-only model (Figure 1) and underscore the value of combining AI-derived features, mtRNA data, and classical clinical predictors in NSCLC.

### Survival analysis based on the combined model

**FIGURE 4:**
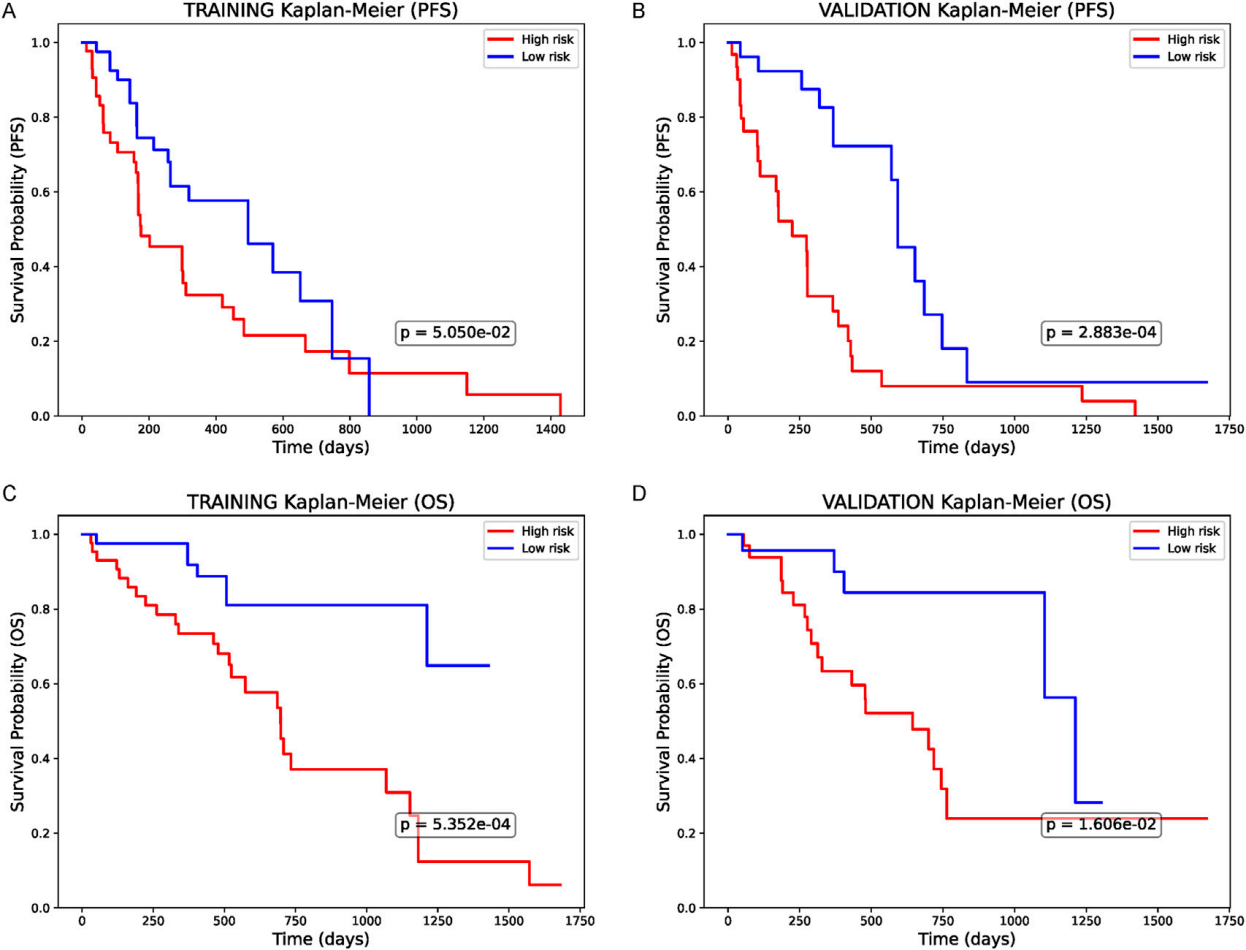
Survival Analysis Based on the Combined Predictor **(A)** Kaplan-Meier curve for progression-free survival (PFS) in the training cohort, stratified by the integrated model’s risk score (clinical + AI + mtRNA). High-risk patients demonstrate significantly worse PFS (p < 0.05). **(B)** PFS in the validation cohort, similarly, showing a notable difference between high- and low-risk groups (p < 0.05). **(C)** Overall survival (OS) in the training set reveals significantly shorter survival among the high-risk group (p < 0.05). **(D)** OS in the validation set, confirming the prognostic value of this combined risk score (p < 0.05). All p-values are based on log-rank tests.

Lastly, we converted the combined predictor (clinical + AI + mtRNA, i.e., Model B) into a single risk score and stratified patients into high- and low-risk groups. In Kaplan-Meier curves for progression-free survival (PFS), high-risk patients in the training cohort (Figure 4A) showed significantly worse outcomes (p < 0.05) than their low-risk counterparts, and an even more pronounced difference was noted in the validation group (Figure 4B, p = 2.883e−04). A similar trend appeared for overall survival (OS): high-risk individuals in the training set (Figure 4C) experienced significantly shorter survival (p < 0.05), which was confirmed in the validation cohort (Figure 4D, p < 0.05). Overall, these results demonstrate that an integrated risk score incorporating clinical factors, AI-driven features, and mtRNA ratio effectively segregates patients into distinct prognostic groups, supporting its potential utility for chemotherapy decision-making in NSCLC.

## Discussion

The present study investigated an integrative framework that incorporates clinical factors, an AI-based prediction variable (BiomedGPT), and a mitochondria-derived RNA (mtRNA) ratio to enhance the stratification of chemotherapy responses in non-small cell lung cancer (NSCLC). Our key finding was that neither traditional clinical variables nor mtRNA ratio alone provided robust predictive power across both training and validation cohorts, while the BiomedGPT model approached higher performance levels but still benefited substantially from the inclusion of mtRNA data. The final, combined model showed the most reliable predictions for chemotherapy efficacy and successfully identified two prognostically distinct risk groups. These results extend earlier observations that mtRNAs, already suggested to be useful diagnostic markers, may also serve as potential predictive indicators of therapeutic outcomes.

One plausible explanation for the improved performance of the integrated approach is that each data source captures distinct yet complementary aspects of tumor biology (Boehm et al., 2022). Clinical variables reflect patient demographics, pathology, and disease stage, while mtRNAs may reflect metabolic or apoptotic adaptations that influence tumor response to cytotoxic agents. Meanwhile, AI-driven insights from BiomedGPT could reveal complex image-based phenotypes, such as subtle radiomic features, that correlate with therapeutic susceptibility. By integrating these diverse predictors, the model achieves a synergy that transcends single-modality assessments, addressing the inherent heterogeneity of NSCLC. Moreover, the superior AUCs obtained by merging these factors suggest that advanced learning algorithms can effectively integrate multi-dimensional data, offering a more comprehensive biological understanding.

We acknowledge the relatively modest sample size of this study, which, while adequate for proof-of-concept and preliminary modeling, limits the generalizability of the findings. To address this limitation, future studies must include larger, multi-institutional datasets with diverse patient demographics to validate the model’s performance across varied clinical contexts. Despite the small sample size, the observed trends align with prior research, lending support to the validity of the conclusions. We further propose that multi-center collaborations and broader patient cohorts in future studies will refine and validate the generalizability of the integrated approach. To enhance the translational potential of this research, future work must focus on external validation through prospective clinical trials. Additionally, developing user-friendly AI tools that integrate seamlessly into clinical workflows will be critical to adoption. Such tools should allow clinicians to leverage the predictive power of the model without requiring extensive computational expertise. By embedding this framework into real-time clinical decision-support systems, the model could significantly enhance personalized treatment planning and improve patient outcomes.

In conclusion, our approach highlights the benefits of combining traditional clinical features, advanced AI-driven imaging analyses, and mtRNA biomarkers to predict chemotherapy response more accurately in NSCLC. By streamlining the discovery of novel drug targets and refining patient risk stratification, this integrated paradigm exemplifies a practical step toward more personalized, mechanism-focused treatment strategies. Further research should focus on larger-scale prospective trials, mechanistic studies of mtRNA function, and enhanced interpretability frameworks for AI-based models to propel these findings toward clinical translation.

## Data Availability

The raw data supporting the conclusions of this article will be made available by the authors, without undue reservation.

## Supplementary material

SUPPLEMENTARY TABLE S1 Summary of Patient Demographics.
